# Symptomatic, Genetic, and Mechanistic Overlaps between Autism and Alzheimer’s Disease

**DOI:** 10.3390/biom11111635

**Published:** 2021-11-04

**Authors:** Muhammad Shahid Nadeem, Salman Hosawi, Sultan Alshehri, Mohammed M. Ghoneim, Syed Sarim Imam, Bibi Nazia Murtaza, Imran Kazmi

**Affiliations:** 1 Department of Biochemistry, Faculty of Science, King Abdulaziz University, Jeddah 21589, Saudi Arabia; mhalim@kau.edu.sa (M.S.N.); shosawi@kau.edu.sa (S.H.); 2 Department of Pharmaceutics, College of Pharmacy, King Saud University, Riyadh 11451, Saudi Arabia; salshehri1@ksu.edu.sa (S.A.); simam@ksu.edu.sa (S.S.I.); 3 Department of Pharmacy Practice, College of Pharmacy, AlMaarefa University, Ad Diriyah 13713, Saudi Arabia; mghoneim@mcst.edu.sa; 4 Department of Zoology, Abbottabad University of Science and Technology (AUST), Abbottabad 22310, Pakistan; nazia.murtaza@gmail.com

**Keywords:** autism spectrum disorder, Alzheimer’s disease, symptoms, genes, pathogenesis, mechanisms

## Abstract

Autism spectrum disorder (ASD) and Alzheimer’s disease (AD) are neurodevelopmental and neurodegenerative disorders affecting two opposite ends of life span, i.e., childhood and old age. Both disorders pose a cumulative threat to human health, with the rate of incidences increasing considerably worldwide. In the context of recent developments, we aimed to review correlated symptoms and genetics, and overlapping aspects in the mechanisms of the pathogenesis of ASD and AD. Dementia, insomnia, and weak neuromuscular interaction, as well as communicative and cognitive impairments, are shared symptoms. A number of genes and proteins linked with both disorders have been tabulated, including MECP2, ADNP, SCN2A, NLGN, SHANK, PTEN, RELN, and FMR1. Theories about the role of neuron development, processing, connectivity, and levels of neurotransmitters in both disorders have been discussed. Based on the recent literature, the roles of FMRP (Fragile X mental retardation protein), hnRNPC (heterogeneous ribonucleoprotein-C), IRP (Iron regulatory proteins), miRNAs (MicroRNAs), and α-, β0, and γ-secretases in the posttranscriptional regulation of cellular synthesis and processing of APP (amyloid-β precursor protein) have been elaborated to describe the parallel and overlapping routes and mechanisms of ASD and AD pathogenesis. However, the interactive role of genetic and environmental factors, oxidative and metal ion stress, mutations in the associated genes, and alterations in the related cellular pathways in the development of ASD and AD needs further investigation.

## 1. Introduction

Autism spectrum disorder (ASD) and Alzheimer’s disease (AD) are two neuropathological or neuropsychiatric conditions affecting young and old individuals, respectively [1,2]. Represented by hearing/speech impairments, weak immunity, poor cognitive functioning, weak neuromuscular interaction, insomnia, problems in gastrointestinal functioning, and social interaction, ASD is mostly diagnosed in children between the ages of 18 months and 3 years. Currently affecting around 1 in 58 young children, it is one of the most rapidly growing neurological conditions worldwide [3,4]. Both the pathophysiology and etiology of ASD are poorly understood. However, many environmental, genetic, and mother-and-child associated risk factors for ASD have been reported [5,6]. Alzheimer’s disease (AD) is characterized by dementia, cognitive impairment, and memory loss in elderly populations. According to estimates, 5.8 million US citizens aged 65 or above are battling the disease, with this number expected to exceed 88 million in 2050 [7,8]. Accumulating amyloid-β peptide (Aβ), resulting in plaques and microtubular protein tau-causing neurofibrillary tangles, are responsible for AD [9]. Gradual disintegration of synaptic clefts, atrophy of neurons, and dementia have been linked with the aggregation of Aβ and tau. Disease can be categorized on the basis of these proteins [10,11,12]. Recent investigations have highlighted a few coinciding aspects of ASD and AD, two neurological and psychiatric diseases affecting two entirely different age groups. Both ASD and AD have been characterized by dementia, poor cognitive functions, speech impairments, intellectual disability (ID), and depression [13,14,15,16]. The prevalence and severity of both diseases involve complex interaction between the genetic, epigenetic, and environmental factors. Mitochondrial dysfunction and inflammation have also been reported to play an important role in the progression of ASD [17,18]. Studies conducted on 12 genes of Notch and 31 genes of WNT (wingless) signaling pathways associated with AD have shown common pathophysiology of ASD and AD [19]. Activity-dependent neuroprotective protein (ADNP), associated with autophagy, represents another common target for the management of autism, Alzheimer’s disease, and schizophrenia [20]. Recently, the processing mechanism of amyloid-β precursor protein (APP) along the anabolic and catabolic pathways has described the overlapping pathophysiology of ASD and AD [21]. Recently, many correlated medicinal interventions against the common symptoms in both diseases have been adopted. For example, for aggressive and agitated ASD and AD patients, antipsychotics, including quetiapine and risperidone, have been found effective [22,23,24]. For depression and anxiety, SSRIs (selective serotonin reuptake inhibitors), such as venlafaxine and citalopram, have been recommended [24,25,26]. Furthermore, to manage sleep problems and improve neuron health and physiology, melatonin has been found effective [27,28]. On the basis of recent developments, we intend to present a comprehensive account of mechanistic and interventive overlaps between ASD and AD. 

## 2. Methodology

The present review study was conducted at the digital library of King Abdulaziz University, Jeddah Saudi Arabia. There was no need for ethical approval or permission as no animal or human subjects were directly included. Associated literature about the overlapping symptoms, mechanisms, and medical interventions in autism and Alzheimer’s disease was collected. Based on the information available in the recent literature, the commonalities in the pathophysiology of both diseases were described.

Data were gathered from web databases, including Yahoo, Google Scholar, PubMed, and Web of Science. Terms such as autism, Alzheimer’s disease, genes in autism, genes in Alzheimer’s disease antidepressants, antipsychotics, sleep promoters, autism pathogenesis, and Alzheimer’s disease pathogenesis were used to collect data. Recent data were acquired from peer-reviewed research publications published in reputable journals. Data from websites, unpublished articles, and published articles in non-peer reviewed journals were excluded. Data included in the final document were combined, analyzed, and evaluated. Overall, 278 information sources (published articles, books, and websites) were combined, and 230 were included in the present study.

**Figure 1:**
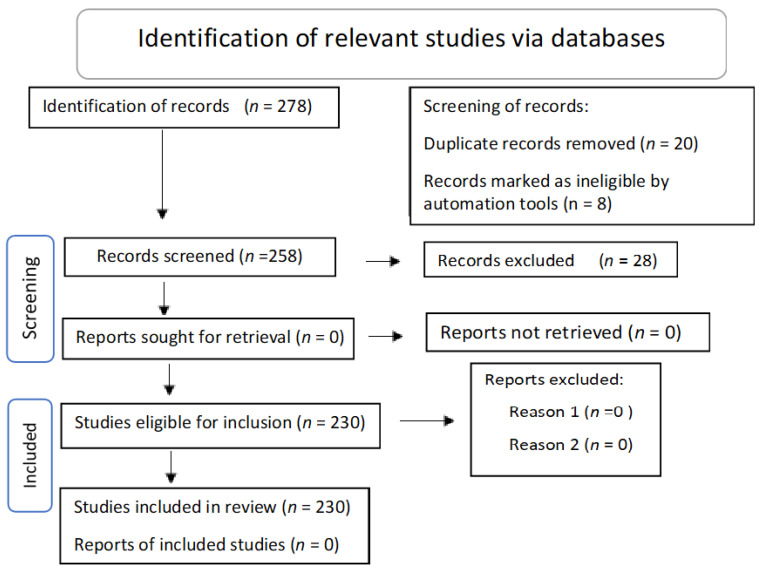
Prisma flow diagram for systematic review.

Prisma flow diagram for systematic review, Figure 1, representing the records identified from all databases searched. The records excluded and included in the present review are described.

## 3. Shared Symptoms of ASD and AD

Both ASD and AD are categorized as neurodevelopmental and neurodegenerative diseases. Several peripheral neuropathies or polyneuropathies that may be the collective effect of mitochondrial disorders have been associated with ASD [29,30,31]. Recently, neurofibromatosis type 1 (NF1) has been associated with the severity of ASD and ADHD (attention-deficit/hyperactivity disorder) [32]. Neuropathologically, AD is caused by the extracellular build-up of amyloid-β (Aβ) plaques and the intracellular accumulation of tau protein, the component of neurofibrillary tangles (NFTs) [33,34]. 

Dementia is a complex term encompassing memory loss, difficulty or inability in reasoning, and diminished problem-solving abilities. Dementia and ASD are closely linked by their symptoms. Many symptoms of dementia, including impairments in social interaction [35], highly complex routines, food selections, and exaggerated response to sensory or physical irritations, are similar to those of ASD [36,37]. Many experts have shown symptomatic identity in dementia and ASD [38]. Recently, in a meta-analysis of 12 studies, researchers declared Alzheimer’s disease the most common type of dementia, although they could not significantly differentiate between AD and dementia [9,39]. Some other studies have considered AD to be one of the major causes of dementia [40]. Hence, dementia is one of the representative overlapping features of AD and ASD. However, further studies are required to delineate between ASD, AD, and dementia.

Most children with autism have very limited language and interaction skills. By contrast, some have very good vocabulary and can discuss a particular topic in detail. However, about 22% of these children lose their vocabulary as they develop [41]. Some children with autism start speaking between the age of 5 and 7, and a few fail to learn a language by the age of 13 years. Early delay in speech and language is more prevalent in male children [42,43]. Alzheimer’s disease is initiated by memory loss, followed by the inability to use language in communication and social interaction. Gradual loss of vocabulary by neurodegeneration is one of the major symptoms of AD that is linked with the loss of cognitive functions [44]. Individuals suffering from AD face more difficulty in denomination tests; they use empty long pauses during discussion. However, the severity of this symptom depends on the stage of disease and is positively correlated with AD progression [45,46,47]. The distribution of speech pauses has been described as a marker for AD diagnosis [48]. Studies have shown that identification of language and diarization of speakers provide promising results for the diagnosis of speech loss in the case of AD [49].

The neurological skills or abilities a person uses to read, remember, concentrate, or make decisions are known as cognitive skills. Having poor cognitive skills is one of the characteristics of children with autism. Some children develop variable cognitive skills with age, but most of them fail to develop a social network [50,51]. The fact that a few develop better cognitive skills compared to other children with autism may be due to compensation mechanisms. According to recent estimates, up to 70% of individuals with ASD have ID (intellectual disability), while only half of those with ID have ASD [52]. Children with symptoms of attention-deficit/hyperactivity disorder (ADHD) along with ASD have shown severe cognitive problems [53]. Generally, a decline in cognitive skills is considered to be a phenomenon of aging, with an increased rate detected after the age of 65 [54,55]. However, this decline in cognitive abilities cannot severely affect normal life activities [56]. Alzheimer’s disease rapidly deteriorates cognitive functions [57]. Plasma D-glutamate levels have been suggested as a marker for the detection of mild cognitive impairments and AD [58].

Anxiety and depression are two major signs of ASD, with up to 84% children with autism having mild to severe anxiety [59,60]. Among autistic children, multiple impairments can be a cause of anxiety. Determination of anxiety levels in children with autism is a difficult process [61,62,63], as the levels increase with age [64,65]. High intensity of anxiety has been reported in children with less severe autism and higher IQ (intelligence quotient) levels [66]. Many strategies adopted by parents to hide autistic traits during social interactions may also increase the level of depression among children with ASD [67]. Alzheimer’s disease is also accompanied by neuropsychiatric symptoms, including depression [68]. According to the meta-analysis of recent studies, up to 16% of AD patients exhibited major depression [69]. AD increases the severity of depression, which in turn induces cognitive impairments [70].

**Figure 2:**
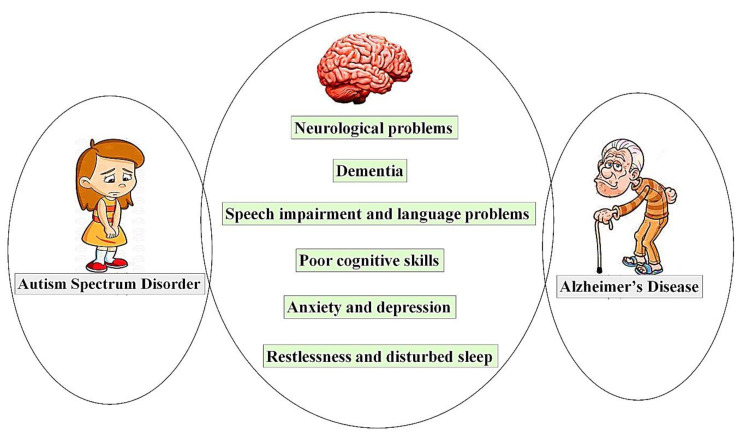
A brief illustration of correlated signs and symptoms representing autism spectrum disorder and Alzheimer’s disease.

Children with autism experience up to an 80% frequency of sleep disturbance, mainly due to a deficiency of melatonin [71,72]. Restlessness is common among children with ASD [73]. Disturbance in circadian rhythms, sleep apnea, and fragmented nocturnal sleep are also among the core symptoms of AD [74]. Circadian rhythm and sleep duration contribute to the behavioral functions and brain development [75,76]. Interventions for sleep disturbances have been suggested to modulate the symptoms of cognitive impairment [77,78]. According to the recent international criteria for the diagnosis of AD, the disease does not necessarily interfere with any occupational or social functioning at early stages. However, gradual impairments in memory and cognitive functions are prominent signs of AD [79]. Similar diagnostic and staging procedures and biomarkers have been used for AD and early memory impairment [80]. Structural disintegration and loss of neuronal connections have recently been identified by magnetic resonance imaging (MRI). Studies have also shown that physical activity may ameliorate the decline in the functioning of white matter and, therefore, AD progression [81]. Some of the correlated symptoms of ASD and AD are illustrated in Figure 2.

## 4. Genes and Genetics

Numerous genes have been linked with the neurological abnormalities such as ASD and AD. According to some studies, 1007 autism risk genes have been reported. Using the SFARI gene database, 212 genes—environment interacting pairs—have been identified [82,83]. Some examples of the genes associated with autism include MECP2, CHD8, KMT2A, GRIN2B, SCN2A, NLGN1, NLGN3, MET, CNTNAP2, FOXP2, TSHZ3, SHANK3, PTEN, DYRK1A, RELN, FOXP1, SYNGAP1, NRXN [84,85], the NLGN gene series (NLGN1, NLGN2, NLGN4, and NLGN4Y) [86,87], brain function protein-related genes (such as GABRA5, GABRB3, UBE3A, HERC2 and CYFIP1 [88]), NRXN2, POGZ, RFX3, ANK2, ARHGEF10, BRD3, CEP152, CHRM3 [89], STX1A, NLGN3, SHANK2, DLGAP1, NRXN3, DLG4, CACNG2, AKAP9, CACNA1C, KCNS3, CACNA2D3 [90], and some metabolic genes, (such as GSTT1, GSTM1, and GSTP1 [91]). Many of these genes are pleiotropic in nature [92]. Mostly, these genes regulate many other genes. For example, up to 3% of individuals have fragile X syndrome (FXS) caused by mutations in the FMR1 gene—a gene that regulates about 6000 mRNAs in the brain and maintains synaptic plasticity [93]. A set of 805 DNA methylation regions (DMRs) of paternal sperm were demonstrated to predict paternal susceptibility to autism in their offspring [94]. Rett syndrome is another disorder associated with ASD. It mostly occurs in females and is caused by mutations in the MECP2 gene, which regulates several genes in neurons [95]. Williams syndrome and autism have shown a similar pattern of gene expression but different socio-cognitive profiles [96].

Variants of at least three dominant autosomal genes have been reported in Alzheimer’s disease. These genes, which are found responsible for the early onset of AD, include presenilin 1 (PSEN1), presenilin 2 (PSEN2), and amyloid precursor protein (APP), and variations in these genes are clearly associated with the early onset of AD [97,98]. As these genes are autosomal dominant, the disease is generationally inherited. Almost 50% of the children of affected patients have the chance to adopt the mutated allele. Mutations in these three genes result in the enhanced production of AB (amyloid beta) fragments and plaque formation [99]. Polymorphic variations in the gene coding for apolipoprotein E (APOE) results in the formation of three isoforms of protein. However, these variants are not found to be critically involved in the development of AD, as they have 8% to 77% frequency in normal human populations worldwide [100,101]. Some genetic factors associated with ASD and AD are tabulated (Table 1).

## 5. Theories and Mechanism of Pathophysiology

Autism is a neurodevelopmental disease affecting very young children, while Alzheimer’s disease is a neurodegenerative disease affecting elderly people. AD is associated with the shrinking of the amygdala, whereas ASD is represented by an enlarged amygdala region. The association between neurodevelopmental and neurodegenerative diseases is a topic of growing interest. In this section, we discuss some theories and mechanisms to emphasize the correlation or overlaps between these apparently divergent conditions.

### 5.1. Disrupted Neural Connectivity

In the neurotypical brain, old, unwanted, and non-functional neurons are removed to improve the networking and operational efficacy of neural circuits. This ability is missing in ASD children and results in poor interaction between neurons and also between the different regions of the brain. In the case of autism, there is an extensive increase in the number of neurons, resulting in the poor shape and fine-tuning of neurological circuits [181,182,183,184,185]. In the autistic brain, there are flaws in the process of removing poorly functional synapses, resulting in overall dented synaptogenesis with abnormal dendritic spines that hinder the neural coordination. Rett syndrome, which presents this condition, is associated with ASD [186,187]. Rett syndrome accounts for less than 1% of ASD, occurring almost exclusively in girls, while autism is diagnosed four times more frequently in boys. The mTORC1 (mechanistic target of rapamycin (mTOR) complex 1) pathway is differentially regulated in Rett syndrome and other syndromes associated with ASD [188]. In the case of AD, the abnormal accumulation of soluble Aβ results in the impairment of neural circuits and increases the number of hyperactive cells [189]. According to relevant studies, the normal level of soluble Aβ supports synaptic plasticity, while increased levels trigger a toxic cascade that results in synaptic impairment and cognitive deficits [190]. Hence, in both cases, neural connectivity and synaptic structure and physiology are responsible for disturbed neural and brain functions.

### 5.2. Imbalanced Neurotransmitters

Elevated serotonin (5-hydroxytryptamine, 5-HT) levels (hyperserotonemia) have been found in more than 30% of children with autism as compared to normal controls. About 99% of blood serotonin is found in the platelets and 1% in the plasma exposed to catalytic enzymes [191]. Serotonin plays an important role in brain development in younger populations and in maintaining proper physiology in adults, as well as in regulating autonomic, behavioral, and cognitive functions [192]. During pregnancy, serotonin is produced by the placenta from tryptophan obtained from the mother, with the brain beginning its synthesis later on [193]. Variations in the genes that regulate the mechanism of serotonin production lead to abnormal levels in the blood as well as neurodevelopmental abnormalities in the CNS (central nervous system) of patients. On the other hand, reduced levels of serotonin have been found in many areas of the brain in AD patients. Serotoninergic system problems have been implicated in anxiety, depression, aggression, and restlessness in AD patients [194].

Dopamine is one of the most extensively studied neurotransmitters, with an established role in neurological and psychological disorders [195]. According to recent findings, brain dysfunction in autism is associated with overactivated dopamine systems. First, there is the link between hyperdopaminergic activity and autism; second, there is the link between right-hemispheric dysfunction and deficiencies in autism; and third, dopamine activation is associated with an increase in the prenatal and perinatal risk factors that increase the incidence of autism-associated behavioral traits [5]. Emotion processing control in the amygdala is inhibited by the activated dopamine system [196,197]. A meta-analysis of seventeen studies and 512 patients has shown a decreased level of dopamine in AD patients as compared to normal controls, indicating a positive association between decreased dopamine levels and the pathophysiology of AD [198]. Despite being a major neurotransmitter, dopamine has recently been named as a factor in the onset of AD [199]. Glutamate is one of the main neurotransmitters, and iGluRs (ligand-gated ionotropic glutamate receptors) play a crucial role in synaptic plasticity and memory. In the cerebral cortex, glutamatergic neurons comprise up to 80% of the brain’s total metabolic activity under non-stimulated conditions [200,201]. Disruption of iGluRs has been associated with neuropathological conditions, such as Alzheimer’s disease and brain damage. It has also been demonstrated that iGluRs and their regulatory proteins are also altered in ASD and fragile X syndrome [202,203].

### 5.3. Overlapping Mechanisms of Pathogenesis

**Figure 3:**
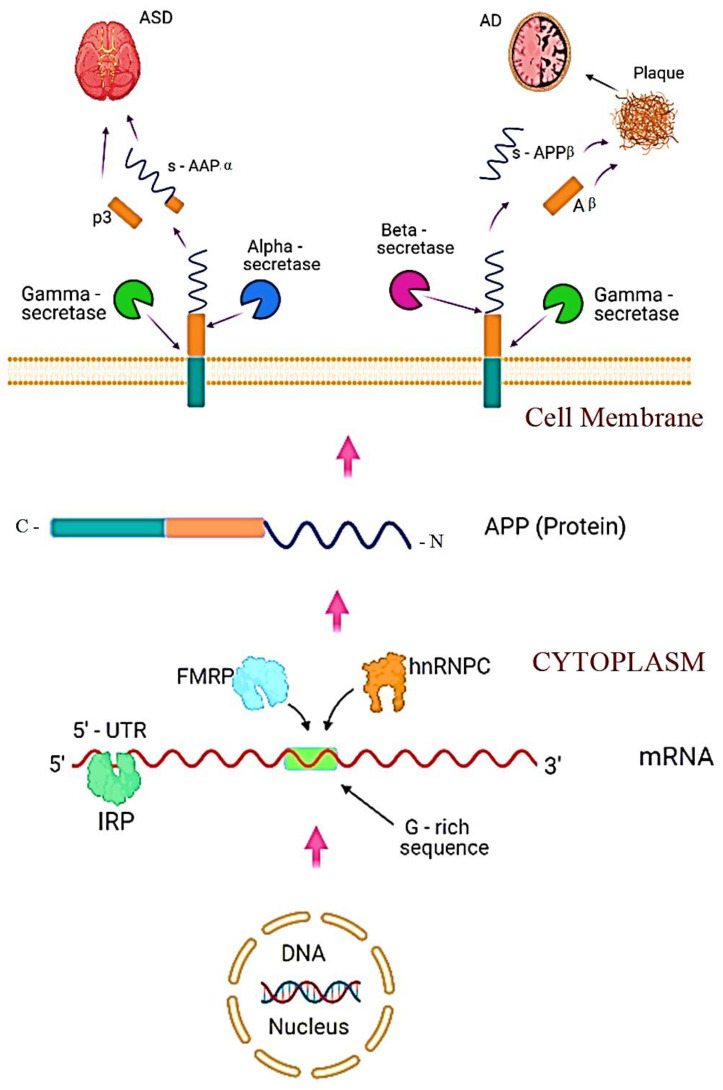
Parallel yet divergent proposed mechanism of ASD and AD pathogenesis. Transport and translation of APP mRNA are well regulated by mRNA transport proteins and RNA binding proteins, such as IRPs and FMRP and miRNAs. There is a G-rich domain in the coding region of mRNA. FMRP and hnRNPC compete to bind this domain to suppress or enhance translation, respectively. APP is a transmembrane protein that is processed by combinations of α-, β-, and γ-secretases, resulting in different product combinations. p3 and s-AAPα promote ASD downstream, while s-APPβ and Aβ, in combination with other molecules, result in the formation of senile plaques and cause AD.

Autism and Alzheimer’s disease (AD) represent two specific yet correlated disorders. Each of them appears at opposite ends of the human lifespan (early and old age; ASD is represented by an enlarged amygdala, while AD is linked with reduced amygdala); ASD is neurotrophic and AD is neurodegenerative in nature [21,204,205]. Many proteins have been reported to contribute to the pathophysiology of ASD and AD [206]. Mutated or non-functional brain protein SHANK3 is responsible for communication problems in children with autism [207,208]. Downregulation of SHANK3 has also been linked with AD [209]. Fragile X mental retardation protein (FMRP) [164,210,211] and heterogeneous ribonucleoprotein-C (hnRNPC) have also been linked with both diseases [212]. Some RNA binding proteins, including IRP-1 and IL-1, also contribute to the pathogenesis of these diseases [21,213]. However, beta-amyloid precursor protein (APP) plays a central role in the pathophysiology of both ASD and AD [204,214]. According to recent information, the pathogenesis of ASD and AD proceeds in parallel in the context of the synthesis and processing of β-amyloid precursor protein (APP). In general, the synthesis of APP is regulated by several factors. At post-transcriptional levels, many types of miRNAs downregulate protein synthesis. Alterations in these miRNAs have been found in AD, and such miRNAs are a topic of considerable importance in management of the disease [215,216,217]. Iron regulatory proteins (IRPs) bind to the 5′-UTR of mRNA and can regulate the translation in an iron-dependent manner, i.e., by promoting protein synthesis in cells with sufficient iron contents and vice versa [218,219]. FMRP is an RNA-binding protein abundantly produced in brain, and its reduction leads to the onset of fragile X syndrome. The protein binds the APP mRNA in the guanine-rich coding region and represses translation and APP synthesis [220]. FMRP also regulates the export of APP mRNA from the nucleus [216,221]. Another RNA binding protein, heterogeneous nuclear ribonucleoprotein C (hnRNPC), competes with FMRP for the mRNA binding site and upregulates APP synthesis [220,222]. Hence, hnRNPC and FMRP regulate protein synthesis in opposite directions. Therefore, a crosstalk between hnRNPC and FMRP should be present for appropriate homeostasis of APP in the different regions of the brain. FMRP also regulates the cytoplasmic level of α-secretase and γ-secretase and decides the fate of APP processing towards catabolic or anabolic pathways, resulting in AD or ASD, respectively [21,223,224]. The digestion of APP with α-secretase and γ- secretase initiates a non-amyloidogenic pathway, resulting in the production of p3 and s-APPα protein moieties. sAPPα (soluble amyloid precursor protein) persuades an upregulation of glutamatergic synapses and a decline in GABAergic synapses, which results in an excitatory/inhibitory imbalance as found in the case of ASD. Additionally, sAPPα overturns the GABAergic synapses by reducing the release of presynaptic vesicles by direct binding of the sAPP extension domain to GABA type B [225,226]. FMRP and hnRNPC regulate the imbalance between total secreted APP (s-APPα), and Aβ indicates a disturbed iron balance, disrupted neuron density, and inter-neuron transmission, resulting in autism [227,228]. Although the elevated level of s-AAPα is associated with autism, an overall increase in the synthesis of APP is not found in autism [229]. Overgrowth of both white and gray matter in the brain, resulting in an enlarged brain size at an early age in children with autism, is based on neurotrophic activity caused by s-APPα and associated factors [230]. Based on the information available, a parallel yet divergent mechanism of ASD and AD pathogenesis is proposed (Figure 3).

## 6. Conclusions

ASD and AD are neurodevelopmental and neurodegenerative disorders affecting two opposite ends of the human life span, i.e., young children and old people, respectively. Many common symptoms, including speech and language problems, cognitive issues, dementia, reasoning, and planning disabilities, have been observed in both disorders. The common genetic links and genes involved in the pathogenesis of both diseases have been tabulated. However, the pathways involved in the pathophysiology of ASD and AD and the effects of specific gene mutations on the development of these diseases need further investigation. The common factors and physiological conditions have been discussed in detail, and overlapping mechanisms of pathogenesis for both diseases have been proposed. The interaction of genetic and environmental factors, including iron imbalance, oxidative stress, inflammatory cytokines, and their qualitative and quantitative role in the pathophysiology of both disorders also need further study.

## Figures and Tables

**Table 1:** Shared genes and factors associated with ASD and AD.

Sr. No.	Genes Associated with ASD and AD	Reference
1	MECP2 (methyl-CpG binding protein 2)	[102,103].
2	ADNP (activity-dependent neuroprotective protein)	[20,104].
3	GRIN2B (glutamate ionotropic receptor NMDA type subunit 2B)	[105,106,107].
4	SCN2A (sodium voltage-gated channel alpha subunit 2)	[108,109].
5	NLGN (neuroligin)	[110,111,112].
6	CNTNAP2 (contactin-associated protein 2)	[113,114,115,116].
7	TSHZ3 (teashirt zinc finger homeobox 3)	[117,118,119].
8	SHANK	[120,121,122,123,124].
9	PTEN (phosphatase and tensin homolog)	[125,126,127,128].
10	DYRK1A (dual-specificity tyrosine phosphorylation-regulated kinase 1A)	[129,130,131,132].
11	RELN (reelin)	[133,134,135].
12	FOXP1 (forkhead box protein P1)	[136,137,138,139].
13	SYNGAP1 (synaptic Ras GTPase-activating protein 1)	[140,141].
14	GABRA5 (gamma-aminobutyric acid type A receptor subunit alpha5)	[142,143].
15	UBE3A (ubiquitin-protein ligase E3A)	[144,145,146].
16	CYFIP1 (cytoplasmic FMR1-interacting protein 1)	[147,148].
17	NRXN (neurexin)	[149,150].
18	STX1A (syntaxin 1A)	[151,152,153].
19	DLG4 (discs large MAGUK scaffold protein 4)	[154,155,156].
20	AKAP9 (A-kinase anchoring protein 9)	[157,158].
21	CACNA2D3 (calcium voltage-gated channel auxiliary subunit alpha2delta 3)	[159,160,161].
22	GSTT1(glutathione S-transferase theta-1), GSTM1(glutathione S-transferase Mu 1), GSTP1(glutathione S-transferase pi 1)	[162,163].
23	FMR1 (fragile X mental retardation 1)	[164,165,166].
24	APOE (apolipoprotein E)	[167,168,169,170].
25	APP (beta-amyloid precursor protein)	[171,172].
26	BDNF (brain-derived neurotrophic factor)	[173,174,175,176].
27	TNF (tumor necrosis factor)	[177,178].
28	SLC6A4 (solute carrier family 6 (neurotransmitter transporter, serotonin), member 4)	[179,180].

## Data Availability

Not applicable.

## References

[1] Varghese M., Keshav N., Jacot-Descombes S., Warda T., Wicinski B., Dickstein D.L., Harony-Nicolas H., De Rubeis S., Drapeau E., Buxbaum J.D. (2017). Autism spectrum disorder: Neuropathology and animal models. Acta Neuropathol..

[2] DeTure M.A., Dickson D.W. (2019). The neuropathological diagnosis of Alzheimer’s disease. Mol. Neurodegener..

[3] Nadeem M.S., Murtaza B.N., Al-Ghamdi M.A., Ali A., Zamzami M.A., Khan J.A., Ahmad A., Rehman M.U., Kazmi I. (2021). Autism-A Comprehensive Array of Prominent Signs and Symptoms. Curr. Pharm. Des..

[4] Dickinson A., Daniel M., Marin A., Gaonkar B., Dapretto M., McDonald N.M., Jeste S. (2021). Multivariate neural connectivity patterns in early infancy predict later autism symptoms. Biol. Psychiatry Cogn. Neurosci. Neuroimaging.

[5] Nadeem M.S., Al-Abbasi F.A., Kazmi I., Murtaza B.N., Zamzami M.A., Kamal M.A., Arif A., Afzal M., Anwar F. (2020). Multiple risk factors: A challenge in the management of Autism. Curr. Pharm. Des..

[6] Katz J., Reichenberg A., Kolevzon A. (2021). Prenatal and perinatal metabolic risk factors for autism: A review and integration of findings from population-based studies. Curr. Opin. Psychiatry.

[7] Soysal P., Tan S.G. (2021). The prevalence and co-incidence of geriatric syndromes in older patients with early-stage Alzheimer’s disease and dementia with Lewy bodies. Aging Clin. Exp. Res..

[8] Rajan K.B., Weuve J., Barnes L.L., McAninch E.A., Wilson R.S., Evans D.A. (2021). Population estimate of people with clinical Alzheimer’s disease and mild cognitive impairment in the United States (2020–2060). Alzheimers Dement..

[9] Lei P., Ayton S., Bush A.I. (2021). The essential elements of Alzheimer’s disease. J. Biol. Chem..

[10] Mintun M.A., Lo A.C., Duggan Evans C., Wessels A.M., Ardayfio P.A., Andersen S.W., Shcherbinin S., Sparks J., Sims J.R., Brys M. (2021). Donanemab in early Alzheimer’s disease. N. Engl. J. Med..

[11] Arnsten A.F., Datta D., Del Tredici K., Braak H. (2021). Hypothesis: Tau pathology is an initiating factor in sporadic Alzheimer’s disease. Alzheimers Dement..

[12] Pereira J.B., Janelidze S., Smith R., Mattsson-Carlgren N., Palmqvist S., Teunissenm C.E., Zetterberg H., Stomrud E., Ashton N.J., Blennow K. (2021). Plasma GFAP is an early marker of amyloid-β but not tau pathology in Alzheimer’s disease. Brain.

[13] Johansson M., Stomrud E., Insel P.S., Leuzy A., Johansson P.M., Smith R., Ismail Z., Janelidze S., Palmqvist S., van Westen D. (2021). Mild behavioral impairment and its relation to tau pathology in preclinical Alzheimer’s disease. Transl. Psychiatry.

[14] Mueller K.D., Hermann B., Mecollari J., Turkstra L.S. (2018). Connected speech and language in mild cognitive impairment and Alzheimer’s disease: A review of picture description tasks. J. Clin. Exp. Neuropsychol..

[15] Rhodus E.K., Barber J., Abner E.L., Duff D., Bardach S.H., Caban-Holt A., Lightner D., Rowles G.D., Schmitt F.A., Jicha G.A. (2020). Behaviors characteristic of autism spectrum disorder in a geriatric cohort with mild cognitive impairment or early dementia. Alzheimer Dis. Assoc. Disord..

[16] Reeves S., Williams V., Costela F.M., Palumbo R., Umoren O., Christopher M.M., Blacker D., Woods R.L. (2020). Narrative video scene description task discriminates between levels of cognitive impairment in Alzheimer’s disease. Neuropsychology.

[17] Gevezova M., Sarafian V., Anderson G., Maes M. (2020). Inflammation and mitochondrial dysfunction in autism spectrum disorder. CNS Neurol. Disord.-Drug Targets.

[18] Yoo S.M., Park J., Kim S.H., Jung Y.K. (2020). Emerging perspectives on mitochondrial dysfunction and inflammation in Alzheimer’s disease. BMB Rep..

[19] Zeidán-Chuliá F., de Oliveira B.H., Salmina A. (2014). Altered expression of Alzheimer’s disease-related genes in the cerebellum of autistic patients: A model for disrupted brain connectome and therapy. Cell Death Dis..

[20] Sragovich S., Merenlender-Wagner A., Gozes I. (2017). ADNP plays a key role in autophagy: From autism to schizophrenia and Alzheimer’s disease. Bioessays.

[21] Lahiri D.K., Maloney B., Wang R., Sokol D.K., Rogers J.T., Westmark C.J. (2021). How autism and Alzheimer’s disease are TrAPPed. Mol. Psychiatry.

[22] de Freitas Oliveira L., Camargos E.F., Martini L.L., Machado F.V., Novaes M.R. (2021). Use of psychotropic agents to treat agitation and aggression in Brazilian patients with Alzheimer’s disease: A naturalistic and multicenter study. Psychiatry Res..

[23] Im D.S. (2021). Treatment of aggression in adults with autism spectrum disorder: A review. Harv. Rev. Psychiatry.

[24] D’Alò G.L., De Crescenzo F., Amato L., Cruciani F., Davoli M., Fulceri F., Minozzi S., Mitrova Z., Morgano G.P., Nardocci F. (2021). Impact of antipsychotics in children and adolescents with autism spectrum disorder: A systematic review and meta-analysis. Health Qual. Life Outcomes.

[25] Nanjappa M.S., Voyiaziakis E., Pradhan B., Thippaiah S.M. (2020). Use of selective serotonin and norepinephrine reuptake inhibitors (SNRIs) in the treatment of autism spectrum disorder (ASD), comorbid psychiatric disorders and ASD-associated symptoms: A clinical review. CNS Spectr..

[26] Leshem R., Bar-Oz B., Diav-Citrin O., Gbaly S., Soliman J., Renoux C., Matok I. (2021). Selective Serotonin Reuptake Inhibitors (SSRIs) and Serotonin Norepinephrine Reuptake Inhibitors (SNRIs) During Pregnancy and the Risk for Autism spectrum disorder (ASD) and Attention deficit hyperactivity disorder (ADHD) in the Offspring: A True Effect or a Bias? A Systematic Review & Meta-Analysis. Curr. Neuropharmacol..

[27] Gunata M., Parlakpinar H.A., Acet H.A. (2020). Melatonin: A review of its potential functions and effects on neurological diseases. Rev. Neurol..

[28] Lalanne S., Fougerou-Leurent C., Anderson G.M., Schroder C.M., Nir T., Chokron S., Delormem R., Claustrat B., Bellissant E., Kermarrec S. (2021). Melatonin: From Pharmacokinetics to Clinical Use in Autism Spectrum Disorder. Int. J. Mol. Sci..

[29] Frye R.E., Rossignol D.A. (2011). Mitochondrial dysfunction can connect the diverse medical symptoms associated with autism spectrum disorders. Pediatric Res..

[30] Luigetti M., Sauchelli D., Primiano G. (2016). Peripheral neuropathy is a common manifestation of mitochondrial diseases: A single-centre experience. Eur. J. Neurol..

[31] Luigetti M., Primiano G., Cuccagna C. (2018). Small fibre neuropathy in mitochondrial diseases explored with sudoscan. Clin. Neurophysiol..

[32] Morotti H., Mastel S., Keller K., Barnard R.A., Hall T., O’Roak B.J., Fombonne E. (2021). Autism and attention-deficit/hyperactivity disorders and symptoms in children with neurofibromatosis type 1. Dev. Med. Child Neurol..

[33] Jansen I.E., Savage J.E., Watanabe K., Bryois J., Williams D.M., Steinberg S., Sealock J., Karlsson I.K., Hägg S., Athanasiu L. (2019). Genome-wide meta-analysis identifies new loci and functional pathways influencing Alzheimer’s disease risk. Nat. Genet..

[34] Kumar D., Sharma A., Sharma L. (2020). A comprehensive review of Alzheimer’s association with related proteins: Pathological role and therapeutic significance. Curr. Neuropharmacol..

[35] American Psychiatric Association (2013). Diagnostic and Statistical Manual of Mental Disorders.

[36] Ikeda M., Brown J., Holland A.J., Fukuhara R., Hodges J.R. (2002). Changes in appetite, food preference, and eating habits in frontotemporal dementia and Alzheimer’s disease. J. Neurol. Neurosurg. Psychiatry.

[37] Shigenobu K., Ikeda M., Fukuhara R., Maki N., Hokoichi K., Nebu A. (2002). The stereotypy rating inventory for frontotemporal lobar degeneration. Psychiatry Res..

[38] Sakuta S., Hashimoto M., Ikeda M., Koyama A., Takasaki A., Hotta M., Fukuhara R., Ishikawa T., Yuki S., Miyagawa Y. (2021). Clinical features of behavioral symptoms in patients with semantic dementia: Does semantic dementia cause autistic traits?. PLoS ONE.

[39] Aschwanden D., Strickhouser J.E., Luchetti M., Stephan Y., Sutin A.R., Terracciano A. (2021). Is personality associated with dementia risk? A meta-analytic investigation. Ageing Res. Rev..

[40] Sotiropoulos I., Catania C., Pinto L.G., Silva R., Pollerberg G.E., Takashima A., Sousa N., Almeida O.F. (2011). Stress acts cumulatively to precipitate Alzheimer’s disease-like tau pathology and cognitive deficits. J. Neurosci..

[41] Castillo M.A., Urdaneta K.E., Semprún-Hernández N., Brigida A.L., Antonucci N., Schultz S., Siniscalco D. (2019). Speech-stimulating substances in autism spectrum disorders. Behav. Sci..

[42] Tager-Flusberg H., Kasari C. (2013). Minimally verbal school-aged children with autism spectrum disorder: The neglected end of the spectrum. Autism Res..

[43] Reindal L., Nærland T., Weidle B., Lydersen S., Andreassen O.A., Sund A.M. (2021). Structural and Pragmatic Language Impairments in Children Evaluated for Autism Spectrum Disorder (ASD). J. Autism Dev. Dis..

[44] Matias-Guiu J.A., García-Ramos R. (2013). Primary progressive aphasia: From syndrome to disease. Neurología.

[45] Weintraub S., Wicklund A.H., Salmon D.P. (2012). The neuropsychological profile of Alzheimer disease. Cold Spring Harb. Perspect. Med..

[46] Laske C., Sohrabi H.R., Frost S.M., López-de-Ipiña K., Garrard P., Buscema M., Dauwels J., Soekadar S.R., Mueller S., Linnemann C. (2015). Innovative diagnostic tools for early detection of Alzheimer’s disease. Alzheimers Dement..

[47] Meilan J.J., Martinez-Sanchez F., Carro J., Carcavilla N., Ivanova O. (2018). Voice markers of lexical access in mild cognitive impairment and Alzheimer’s disease. Curr. Alzheimer Res..

[48] Pastoriza-Dominguez P., Torre I.G., Dieguez-Vide F., Gomez-Ruiz I., Gelado S., Bello-Lopez J., Avila-Rivera A., Matias-Guiu J., Pytel V., Hernandez-Fernandez A. (2021). Speech pause distribution as an early marker for Alzheimer’s disease. medRxiv.

[49] Campbell E.L., Mesía R.Y., Docío-Fernández L., García-Mateo C. (2021). Paralinguistic and linguistic fluency features for Alzheimer’s disease detection. Comput. Speech Lang..

[50] Nyrenius J., Billstedt E. (2019). The functional impact of cognition in adults with autism spectrum disorders. Nordic J. Psychiatry.

[51] Kenny L., Cribb S.J., Pellicano E. (2019). Childhood Executive Function Predicts Later Autistic Features and Adaptive Behavior in Young Autistic People: A 12-Year Prospective Study. J. Abnorm. Child Psychol..

[52] Bertelli M.O., Keller R. (2019). ASD and Intellectual Disability. Psychopathology in Adolescents and Adults with Autism Spectrum Disorders.

[53] Livingston L.A., Happé F. (2017). Conceptualising compensation in neurodevelopmental disorders: Reflections from autism spectrum disorder. Neurosci. Biobehav. Rev..

[54] Mansour R., Ward A.R., Lane D.M., Loveland K.A., Aman M.G., Jerger S., Schachar R.J., Pearson D.A. (2021). ADHD severity as a predictor of cognitive task performance in children with Autism Spectrum Disorder (ASD). Res. Dev. Disabil..

[55] Cornelis M.C., Wang Y., Holland T., Agarwal P., Weintraub S., Morris M.C. (2019). Age and cognitive decline in the UK Biobank. PLoS ONE.

[56] Malpetti M., Kievit R.A., Passamonti L., Jones P.S., Tsvetanov K.A., Rittman T., Mak E., Nicastro N., Bevan-Jones W.R., Su L. (2020). Microglial activation and tau burden predict cognitive decline in Alzheimer’s disease. Brain.

[57] Marshall G.A., Rentz D.M., Frey M.T., Locascio J.J., Johnson K.A., Sperling R.A. (2011). Alzheimer’s Disease Neuroimaging Initiative. Executive function and instrumental activities of daily living in mild cognitive impairment and Alzheimer’s disease. Alzheimers Dement..

[58] Alzheimer Association (2019). Early Signs and Symptoms of Alzheimer’s Alzheimer’s and Dementia.

[59] Chang C.H., Lin C.H., Liu C.Y., Huang C.S., Chen S.J., Lin W.C., Yang H.T., Lane H.Y. (2021). Plasma d-glutamate levels for detecting mild cognitive impairment and Alzheimer’s disease: Machine learning approaches. J. Psychopharmacol..

[60] Wijnhoven L.A., Creemers D.H., Vermulst A.A. (2018). Prevalence and risk factors of anxiety in a clinical Dutch sample of children with an autism spectrum disorder. Front Psychiatry.

[61] McVey A.J. (2019). The neurobiological presentation of anxiety in autism spectrum disorder: A systematic review. Autism Res..

[62] Kerns C.M., Kendall P.C. (2012). The presentation and classification of anxiety in autism spectrum disorder. Clin. Psychol..

[63] Cervantes P., Matson J.L., Tureck K. (2013). The relationship of comorbid anxiety symptom severity and challenging behaviors in infants and toddlers with autism spectrum disorder. Res. Autism Spectr. Disord..

[64] Vasa R.A., Carroll L.M., Nozzolillo A.A. (2014). A systematic review of treatments for anxiety in youth with autism spectrum disorders. J. Autism Dev. Disord..

[65] Davis III T.E., Hess J.A., Moree B.N. (2011). Anxiety symptoms across the lifespan in people diagnosed with autistic disorder. Res. Autism Spectr. Disord..

[66] Uljarević M., Hedley D., Rose-Foley K., Magiati I., Cai R.Y., Dissanayake C., Richdale A., Trollor J. (2019). Anxiety and depression from adolescence to old age in autism spectrum disorder. J. Autism Dev. Dis..

[67] Salazar F., Baird G., Chandler S. (2015). Co-occurring psychiatric disorders in preschool and elementary school-aged children with autism spectrum disorder. J. Autism Dev. Disord..

[68] Hull L., Levy L., Lai M.C., Petrides K.V., Baron-Cohen S., Allison C., Smith P., Mandy W. (2021). Is social camouflaging associated with anxiety and depression in autistic adults?. Mol. Autism..

[69] Agüera- L., García-Ramos R., Grandas F.J., López-Álvarez J., Rodríguez J.M.M., Rodríguez F.J.O., Olivera Pueyo J., Pelegrín Valero C., Porta-Etessam J. (2021). Depression in Alzheimer’s disease: A Delphi consensus on etiology, risk factors, and clinical management. Front. Psychiatry.

[70] Asmer M.S., Kirkham J., Newton H., Ismail Z., Elbayoumi H., Leung R.H., Seitz D.P. (2018). Meta-analysis of the prevalence of major depressive disorder among older adults with dementia. J. Clin. Psychiatry.

[71] Starkstein S.E., Jorge R., Mizrahi R., Robinson R.G. (2005). The construct of minor and major depression in Alzheimer’s disease. Am. J. Psychiatry.

[72] Kotagal S., Broomall E. (2012). Sleep in children with autism spectrum disorder. Pediatric Neurol..

[73] Mannion A., Leader G. (2014). Sleep problems in autism spectrum disorder: A literature review. J. Autism Dev. Disord..

[74] Nakagawa A., Hayashi W., Nishio T., Hanawa Y., Aoyagi K., Okajima Y., Iwanami A. (2021). Similarity of subjective symptoms between autism spectrum disorder and attention-deficit/hyperactivity disorder in adults: Preliminary findings. Neuropsychopharmacol. Rep..

[75] Outen J., Spira A., Wanigatunga S., Zipunnikov V., Wu M., Rosenberg P. (2021). Circadian Rhythm Disturbance in Agitation of Alzheimer’s disease. Am. J. Geriatr. Psychiatry.

[76] Cheng W., Rolls E., Gong W., Du J., Zhang J., Zhang X.Y., Li F., Feng J. (2020). Sleep duration, brain structure, and psychiatric and cognitive problems in children. Mol. Psychiatry.

[77] Lee S.B., Park J., Kwak Y., Park Y.U., Nhung T.T., Suh B.K., Woo Y., Suh Y., Cho E., Cho S. (2021). Disrupted-in-schizophrenia 1 enhances the quality of circadian rhythm by stabilizing BMAL1. Transl. Psychiatry.

[78] Kent B.A., Feldman H.H., Nygaard H.B. (2021). Sleep and its regulation: An emerging pathogenic and treatment frontier in Alzheimer’s disease. Prog. Neurobiol..

[79] Dubois B., Feldman H.H., Jacova C., Dekosky S.T., Barberger-Gateau P., Cummings J. (2007). Research criteria for the diagnosis of Alzheimer’s disease: Revising the NINCDS-ADRDA criteria. Lancet Neurol..

[80] Dubois B., Feldman H.H., Jacova C., Hampel H., Molinuevo J.L., Blennow K., DeKosky S.T., Gauthier S., Selkoe D., Bateman R. (2014). Advancing research diagnostic criteria for Alzheimer’s disease: The IWG-2 criteria. Lancet Neurol..

[81] Alexiou A., Mantzavinos V.D., Greig N.H., Kamal M.A. (2017). A Bayesian Model for the Prediction and Early Diagnosis of Alzheimer’s disease. Front. Aging Neurosci..

[82] Xiong J., Chen S., Pang N., Deng X., Yang L., He F., Wu L., Chen C., Yin F., Peng J. (2019). Neurological diseases with autism spectrum disorder: Role of ASD risk genes. Front. Neurosci..

[83] Santos J.X., Rasga C., Marques A.R., Martiniano H.F., Asif M., Vilela J., Oliveira G., Vicente A.M. (2019). A role for gene-environment interactions in Autism Spectrum Disorder is suggested by variants in genes regulating exposure to environmental factors. bioRxiv.

[84] Hoffmann A., Spengler D. (2021). Chromatin Remodeler CHD8 in Autism and Brain Development. J. Clin. Med..

[85] Li W., Pozzo-Miller L. (2020). Dysfunction of the corticostriatal pathway in autism spectrum disorders. J. Neurosci Res..

[86] Chen J., Yu S., Fu Y., Li X. (2014). Synaptic proteins and receptors defects in autism spectrum disorders. Front. Cell. Neurosci..

[87] De Rubeis S., Buxbaum J.D. (2015). Genetics and genomics of autism spectrum disorder: Embracing complexity. Hum. Mol. Genet..

[88] Puffenberger E.G., Jinks R.N., Wang H., Xin B., Fiorentini C., Sherman E.A., Degrazio D., Shaw C., Sougnez C., Cibulskis K. (2012). A homozygous missense mutation in HERC2 associated with global developmental delay and autism spectrum disorder. Hum. Mutat..

[89] Li J., Wang L., Guo H. (2017). Targeted sequencing and functional analysis reveal brain-size-related genes and their networks in autism spectrum disorders. Mol. Psychiatry.

[90] Peng J., Zhou Y., Wang K. (2021). Multiplex gene and phenotype network to characterize shared genetic pathways of epilepsy and autism. Sci. Rep..

[91] Rahbar M.H., Samms-Vaughan M., Saroukhani S., Bressler J., Hessabi M., Grove M.L., Shakspeare-Pellington S., Loveland K.A., Beecher C., McLaughlin W. (2021). Associations of Metabolic Genes (GSTT1, GSTP1, GSTM1) and Blood Mercury Concentrations Differ in Jamaican Children with and without Autism Spectrum Disorder. Int. J. Environ. Res. Public Health.

[92] Guo H., Peng Y., Hu Z., Li Y., Xun G., Ou J., Sun L., Xiong Z., Liu Y., Wang T. (2017). Genome-wide copy number variation analysis in a Chinese autism spectrum disorder cohort. Sci. Rep..

[93] Ascano M., Mukherjee N., Bandaru P., Miller J.B., Nusbaum J.D., Corcoran D.L., Langlois C., Munschauer M., Dewell S., Hafner M. (2012). FMRP targets distinct mRNA sequence elements to regulate protein expression. Nature.

[94] Garrido N., Cruz F., Egea R.R., Simon C., Sadler-Riggleman I., Beck D., Nilsson E., Maamar M.B., Skinner M.K. (2021). Sperm DNA methylation epimutation biomarker for paternal offspring autism susceptibility. Clin. Epigenet..

[95] Liu X., Takumi T. (2014). Genomic and genetic aspects of autism spectrum disorder. Biochem. Biophys. Res. Commun..

[96] Niego A., Benítez-Burraco A. (2021). Autism and Williams syndrome: Dissimilar socio-cognitive profiles with similar patterns of abnormal gene expression in the blood. Autism.

[97] Cacace R., Sleegers K., Van Broeckhoven C. (2016). Molecular genetics of early-onset Alzheimer’s disease revisited. Alzheimers Dement..

[98] Hoogmartens J., Hens E., Engelborghs S., De Deyn P.P., van der Zee J., Van Broeckhoven C., Cacace R. (2021). Investigation of the role of matrix metalloproteinases in the genetic etiology of Alzheimer’s disease. Neurobiol. Aging.

[99] Lanoiselée H.M., Nicolas G., Wallon D., Rovelet-Lecrux A., Lacour M., Rousseau S., Richard A.C., Pasquier F., Rollin-Sillaire A., Martinaud O. (2017). APP, PSEN1, and PSEN2 mutations in early-onset Alzheimer disease: A genetic screening study of familial and sporadic cases. PLoS Med..

[100] Han S.H., Hulette C., Saunders A.M., Einstein G., Pericak-Vance M., Strittmatter W.J., Roses A.D., Schmechel D.E. (1994). Apolipoprotein E is present in hippocampal neurons without neurofibrillary tangles in Alzheimer’s disease and in age-matched controls. Exp. Neurol..

[101] Qian J., Wolters F.J., Beiser A., Haan M., Ikram M.A., Karlawish J., Langbaum J.B., Neuhaus J.M., Reiman E.M., Roberts J.S. (2017). APOE-related risk of mild cognitive impairment and dementia for prevention trials: An analysis of four cohorts. PLoS Med..

[102] Wen Z., Cheng T.L., Li G.Z., Sun S.B., Yu S.Y., Zhang Y., Du Y.S., Qiu Z. (2017). Identification of autism-related MECP2 mutations by whole-exome sequencing and functional validation. Mol. Autism.

[103] Li P., Quan W., Wang Z., Chen Y., Zhang H., Zhou Y. (2021). AD7c-NTP Impairs Adult Striatal Neurogenesis by Affecting the Biological Function of MeCP2 in APP/PSl Transgenic Mouse Model of Alzheimer’s Disease. Front. Aging Neurosci..

[104] Ivashko-Pachima Y., Hadar A., Grigg I., Korenková V., Kapitansky O., Karmon G., Gershovits M., Sayas C.L., Kooy R.F., Attems J. (2019). Discovery of autism/intellectual disability somatic mutations in Alzheimer’s brains: Mutated ADNP cytoskeletal impairments and repair as a case study. Mol. Psychiatry.

[105] Andreoli V., De Marco E.V., Trecroci F., Cittadella R., Di Palma G., Gambardella A. (2014). Potential involvement of GRIN2B encoding the NMDA receptor subunit NR2B in the spectrum of Alzheimer’s disease. J. Neural Transm..

[106] Pan Y., Chen J., Guo H., Ou J., Peng Y., Liu Q., Shen Y., Shi L., Liu Y., Xiong Z. (2015). Association of genetic variants of GRIN2B with autism. Sci. Rep..

[107] Hu C., Chen W., Myers S.J., Yuan H., Traynelis S.F. (2016). Human GRIN2B variants in neurodevelopmental disorders. J. Pharmacol. Sci..

[108] Ilyas M., Salpietro V., Efthymiou S., Bourinaris T., Tariq A., Imdad M., Ahmad A., Ahmad H., Houlden H. (2019). Identification of common genetic markers of paroxysmal neurological disorders using a network analysis approach. Neurol. Sci..

[109] Spratt P.W., Ben-Shalom R., Keeshen C.M., Burke K.J., Clarkson R.L., Sanders S.J., Bender K.J. (2019). The autism-associated gene Scn2a contributes to dendritic excitability and synaptic function in the prefrontal cortex. Neuron.

[110] Tristán-Clavijo E., Camacho-Garcia R.J., Robles-Lanuza E., Ruiz A., van der Zee J., Van Broeckhoven C., Hernandez I., Martinez-Mir A., Scholl F.G. (2015). A truncating mutation in Alzheimer’s disease inactivates neuroligin-1 synaptic function. Neurobiol. Aging.

[111] Jamain S., Quach H., Betancur C., Råstam M., Colineaux C., Gillberg I.C., Soderstrom H., Giros B., Leboyer M., Gillberg C. (2003). Mutations of the X-linked genes encoding neuroligins NLGN3 and NLGN4 are associated with autism. Nat. Genet..

[112] Sindi I.A., Dodd P.R. (2015). New insights into Alzheimer’s disease pathogenesis: The involvement of neuroligins in synaptic malfunction. Neurodegener. Dis. Manag..

[113] Alarcón M., Abrahams B.S., Stone J.L., Duvall J.A., Perederiy J.V., Bomar J.M., Sebat J., Wigler M., Martin C.L., Ledbetter D.H. (2008). Linkage, association, and gene-expression analyses identify CNTNAP2 as an autism-susceptibility gene. Am. J. Hum. Genet..

[114] Peñagarikano O., Geschwind D.H. (2012). What does CNTNAP2 reveal about autism spectrum disorder?. Trends Mol. Med..

[115] Hirano A., Ohara T., Takahashi A., Aoki M., Fuyuno Y., Ashikawa K., Morihara T., Takeda M., Kamino K., Oshima E. (2015). A genome-wide association study of late-onset Alzheimer’s disease in a Japanese population. Psychiatry Genet..

[116] Tábuas-Pereira M., Santana I., Guerreiro R., Brás J. (2020). Alzheimer’s disease Genetics: Review of Novel Loci Associated with Disease. Curr. Genet. Med. Rep..

[117] Louwersheimer E., Cohn-Hokke P.E., Pijnenburg Y.A., Weiss M.M., Sistermans E.A., Rozemuller A.J., Hulsman M., van Swieten J.C., van Duijn C.M., Barkhof F. (2017). Rare Genetic Variant in SORL1 May Increase Penetrance of Alzheimer’s Disease in a Family with Several Generations of APOE-ɛ4 Homozygosity. J. Alzheimers Dis..

[118] Caubit X., Gubellini P., Andrieux J., Roubertoux P.L., Metwaly M., Jacq B., Fatmi A., Had-Aissouni L., Kwan K.Y., Salin P. (2016). TSHZ3 deletion causes an autism syndrome and defects in cortical projection neurons. Nat. Genet..

[119] Chabbert D., Caubit X., Roubertoux P.L., Carlier M., Habermann B., Jacq B., Salin P., Metwaly M., Frahm C., Fatmi A. (2019). Postnatal Tshz3 deletion drives altered corticostriatal function and autism spectrum disorder–like behavior. Biol. Psychiatry.

[120] Uchino S., Waga C. (2013). SHANK3 as an autism spectrum disorder-associated gene. Brain Dev..

[121] Mossa A., Giona F., Pagano J., Sala C., Verpelli C. (2018). SHANK genes in autism: Defining therapeutic targets. Prog. Neuro-Psychopharmacol. Biol. Psychiatry.

[122] Durand C.M., Betancur C., Boeckers T.M., Bockmann J., Chaste P., Fauchereau F., Nygren G., Rastam M., Gillberg I.C., Anckarsäter H. (2007). Mutations in the gene encoding the synaptic scaffolding protein SHANK3 are associated with autism spectrum disorders. Nat. Genet..

[123] Zhao Y., Jaber V.R., LeBeauf A., Sharfman N.M., Lukiw W.J. (2019). microRNA-34a (miRNA-34a) mediated down-regulation of the post-synaptic cytoskeletal element SHANK3 in sporadic Alzheimer’s disease (AD). Front. Neurol..

[124] Matas E., Maisterrena A., Thabault M., Balado E., Francheteau M., Balbous A., Galvan L., Jaber M. (2021). Major motor and gait deficits with sexual dimorphism in a Shank3 mutant mouse model. Mol. Autism.

[125] Zhou J., Parada L.F. (2012). PTEN signaling in autism spectrum disorders. Curr. Opin. Neurobiol..

[126] Knafo S., Sánchez-Puelles C., Palomer E., Delgado I., Draffin J.E., Mingo J., Wahle T., Kaleka K., Mou L., Pereda-Perez I. (2016). PTEN recruitment controls synaptic and cognitive function in Alzheimer’s models. Nat. Neurosci..

[127] Frere S., Slutsky I. (2016). Targeting PTEN interactions for Alzheimer’s disease. Nat. Neurosci..

[128] Matsuda S., Nakagawa Y., Tsuji A., Kitagishi Y., Nakanishi A., Murai T. (2018). Implications of PI3K/AKT/PTEN signaling on superoxide dismutases expression and in the pathogenesis of Alzheimer’s disease. Diseases.

[129] Kim O.H., Cho H.J., Han E., Hong T.I., Ariyasiri K., Choi J.H., Hwang K.S., Jeong Y.M., Yang S.Y., Yu K. (2017). Zebrafish knockout of Down syndrome gene, DYRK1A, shows social impairments relevant to autism. Mol. Autism.

[130] van Bon B.W., Coe B.P., Bernier R., Green C., Gerdts J., Witherspoon K., Kleefstra T., Willemsen M.H., Kumar R., Bosco P. (2016). Disruptive de novo mutations of DYRK1A lead to a syndromic form of autism and ID. Mol. Psychiatry.

[131] García-Cerro S., Rueda N., Vidal V., Lantigua S., Martínez-Cué C. (2017). Normalizing the gene dosage of Dyrk1A in a mouse model of Down syndrome rescues several Alzheimer’s disease phenotypes. Neurobiol. Dis..

[132] Smith B., Medda F., Gokhale V., Dunckley T., Hulme C. (2012). Recent advances in the design, synthesis, and biological evaluation of selective DYRK1A inhibitors: A new avenue for a disease modifying treatment of Alzheimer’s?. ACS Chem. Neurosci..

[133] Fehér Á., Juhász A., Pákáski M., Kálmán J., Janka Z. (2015). Genetic analysis of the RELN gene: Gender specific association with Alzheimer’s disease. Psychiatry Res..

[134] Seripa D., Matera M.G., Franceschi M., Daniele A., Bizzarro A., Rinaldi M., Panza F., Fazio V.M., Gravina C., D’Onofrio G. (2008). The RELN locus in Alzheimer’s disease. J. Alzheimers Dis..

[135] Lammert D.B., Howell B.W. (2016). RELN mutations in autism spectrum disorder. Front. Cell. Neurosci..

[136] Chien W.H., Gau S.F., Chen C.H., Tsai W.C., Wu Y.Y., Chen P.H., Shang C.Y., Chen C.H. (2013). Increased gene expression of FOXP1 in patients with autism spectrum disorders. Mol. Autism.

[137] Srinivasan K., Friedman B.A., Etxeberria A., Huntley M.A., Van Der Brug M.P., Foreman O., Paw J.S., Modrusan Z., Beach T.G., Serrano G.E. (2019). Alzheimer’s patient brain myeloid cells exhibit enhanced aging and unique transcriptional activation. BioRxiv.

[138] Garcia-Oscos F., Koch T.M., Pancholi H., Trusel M., Daliparthi V., Park S.E., Ayhan F., Alam D.H., Holdway J.E., Konopka G. (2021). Autism-linked gene FoxP1 selectively regulates the cultural transmission of learned vocalizations. Sci. Adv..

[139] Al-Erjan A.M., Kadhim S., Ahmed M.A., Kadham M.J. (2021). Determine Some Mutations in the Foxp1 Gene in Autistic Patients in Baghdad Governorate. Ann. Rom. Soc. Cell Biol..

[140] Seyfried N.T., Dammer E.B., Swarup V., Nandakumar D., Duong D.M., Yin L., Deng Q., Nguyen T., Hales C.M., Wingo T. (2017). A multi-network approach identifies protein-specific co-expression in asymptomatic and symptomatic Alzheimer’s disease. Cell Syst..

[141] Rolland T., Cliquet F., Anney R.J., Traut N., Mathieu A., Huguet G., Leblond C.S., Douard E., Amsellem F., Malesys S. (2021). Towards a gene-level map of resilience to genetic variants associated with autism. medRxiv.

[142] Lin G., Ji K., Li S., Ma W., Pan X. (2020). The Genetics Analysis of Molecular Pathogenesis for Alzheimer’s Disease. Eur. Neurol..

[143] Mahdavi M., Kheirollahi M., Riahi R., Khorvash F., Khorrami M., Mirsafaie M. (2018). Meta-analysis of the association between GABA receptor polymorphisms and autism spectrum disorder (ASD). J. Mol. Neurosci..

[144] Singh B.K., Vatsa N., Kumar V., Shekhar S., Sharma A., Jana N.R. (2017). Ube3a deficiency inhibits amyloid plaque formation in APPswe/PS1δE9 mouse model of Alzheimer’s disease. Hum. Mol. Genet..

[145] Jason J.Y., Paranjape S.R., Walker M.P., Choudhury R., Wolter J.M., Fragola G., Emanuele M.J., Major M.B., Zylka M.J. (2017). The autism-linked UBE3A T485A mutant E3 ubiquitin ligase activates the Wnt/β-catenin pathway by inhibiting the proteasome. J. Biol. Chem..

[146] Olabarria M., Pasini S., Corona C., Robador P., Song C., Patel H., Lefort R. (2019). Dysfunction of the ubiquitin ligase E3A Ube3A/E6-AP contributes to synaptic pathology in Alzheimer’s disease. Commun. Biol..

[147] Tiwari S.S., Mizuno K., Ghosh A., Aziz W., Troakes C., Daoud J., Golash V., Noble W., Hortobágyi T., Giese K.P. (2016). Alzheimer-related decrease in CYFIP2 links amyloid production to tau hyperphosphorylation and memory loss. Brain.

[148] Wang J., Tao Y., Song F., Sun Y., Ott J., Saffen D. (2015). Common regulatory variants of CYFIP1 contribute to susceptibility for autism spectrum disorder (ASD) and classical autism. Ann. Hum. Genet..

[149] Zheng J.J., Li W.X., Liu J.Q., Guo Y.C., Wang Q., Li G.H., Dai S.X., Huang J.F. (2018). Low expression of aging-related NRXN3 is associated with Alzheimer disease: A systematic review and meta-analysis. Medicine.

[150] Tromp A., Mowry B., Giacomotto J. (2020). Neurexins in autism and schizophrenia-a review of patient mutations, mouse models and potential future directions. Mol. Psychiatry.

[151] Costa A.S., Guerini F.R., Arosio B., Galimberti D., Zanzottera M., Bianchi A., Nemni R., Clerici M. (2019). SNARE complex polymorphisms associate with alterations of visual selective attention in Alzheimer’s Disease. J. Alzheimers Dis..

[152] Tasaki S., Gaiteri C., Mostafavi S., De Jager P.L., Bennett D.A. (2018). The molecular and neuropathological consequences of genetic risk for Alzheimer’s dementia. Front. Neurosci..

[153] Cartier E., Hamilton P.J., Belovich A.N., Shekar A., Campbell N.G., Saunders C., Andreassen T.F., Gether U., Veenstra-Vanderweele J., Sutcliffe J.S. (2015). Rare autism-associated variants implicate syntaxin 1 (STX1 R26Q) phosphorylation and the dopamine transporter (hDAT R51W) in dopamine neurotransmission and behaviors. EBioMedicine.

[154] Coley A.A., Gao W.J. (2018). PSD95: A synaptic protein implicated in schizophrenia or autism?. Prog. Neuro-Psychopharmacol. Biol. Psychiatry.

[155] Liu P., Qian H., Wang L. (2018). Identification of Key Genes Related with Alzheimer’s Disease Treatment Through Bioinformatics Analysis. J. Biol. Life Sci..

[156] Quan X., Liang H., Chen Y.A., Qin Q., Wei Y., Liang Z. (2020). Related network and differential expression analyses identify nuclear genes and pathways in the hippocampus of Alzheimer disease. Medical science monitor: Int. Med. J. Exp. Clin. Res..

[157] Ikezu T., Chen C., DeLeo A.M., Zeldich E., Fallin M.D., Kanaan N.M., Lunetta K.L., Abraham C.R., Logue M.W., Farrer L.A. (2018). Tau phosphorylation is impacted by rare AKAP9 mutations associated with Alzheimer disease in African Americans. J. Neuroimmune Pharmacol..

[158] Poelmans G., Franke B., Pauls D.L., Glennon J.C., Buitelaar J.K. (2013). AKAPs integrate genetic findings for autism spectrum disorders. Transl. Psychiatry.

[159] Villela D., Suemoto C.K., Pasqualucci C.A., Grinberg L.T., Rosenberg C. (2016). Do copy number changes in CACNA2D2, CACNA2D3, and CACNA1D constitute a predisposing risk factor for Alzheimer’s disease?. Front. Genet..

[160] Willsey A.J., State M.W. (2015). Autism spectrum disorders: From genes to neurobiology. Curr. Opin. Neurobiol..

[161] Richard A.E., Scheffer I.E., Wilson S.J. (2017). Features of the broader autism phenotype in people with epilepsy support shared mechanisms between epilepsy and autism spectrum disorder. Neurosci. Biobehav. Rev..

[162] Oshodi Y., Ojewunmi O., Oshodi T.A., Ijarogbe G.T., Ogun O.C., Aina O.F., Lesi F.E. (2017). Oxidative stress markers and genetic polymorphisms of glutathione S-transferase T1, M1, and P1 in a subset of children with autism spectrum disorder in Lagos, Nigeria. Niger. J. Clin. Pract..

[163] Jafarian Z., Saliminejad K., Kamali K., Ohadi M., Kowsari A., Nasehi L., Khorshid K.H.R. (2018). Association of glutathione S-transferases M1, P1 and T1 variations and risk of late-onset Alzheimer’s disease. Neurol. Res..

[164] Hagerman R., Au J., Hagerman P. (2011). FMR1 premutation and full mutation molecular mechanisms related to autism. J. Neurodev. Disord..

[165] Renoux A.J., Carducci N.M., Ahmady A.A., Todd P.K. (2014). Fragile X mental retardation protein expression in Alzheimer’s disease. Front. Genet..

[166] Klusek J., Thurman A.J., Abbeduto L. (2021). Maternal Pragmatic Language Difficulties in the FMR1 Premutation and the Broad Autism Phenotype: Associations with Individual and Family Outcomes. J. Autism Dev. Disord..

[167] Ashley-Koch A.E., Jaworski J., Mei H., Ritchie M.D., Skaar D.A., Delong G.R., Worley G., Abramson R.K., Wright H.H., Cuccaro M.L. (2007). Investigation of potential gene–gene interactions between APOE and RELN contributing to autism risk. Psychiatr. Genet..

[168] Lee E.G., Tulloch J., Chen S., Leong L., Saxton A.D., Kraemer B., Darvas M., Keene C.D., Shutes-David A., Todd K. (2020). Redefining transcriptional regulation of the APOE gene and its association with Alzheimer’s disease. PLoS ONE.

[169] Husain M.A., Laurent B., Plourde M. (2021). APOE and Alzheimer’s disease: From lipid transport to physiopathology and therapeutics. Front. Neurosci..

[170] Hu Z., Yang Y., Zhao Y., Yu H., Ying X., Zhou D., Zhong J., Zheng Z., Liu J., Pan R. (2018). APOE hypermethylation is associated with autism spectrum disorder in a Chinese population. Exp. Ther. Med..

[171] Westmark C.J., Sokol D.K., Maloney B., Lahiri D.K. (2016). Novel roles of amyloid-beta precursor protein metabolites in fragile X syndrome and autism. Mol. Psychiatry.

[172] Jonsson T., Atwal J.K., Steinberg S., Snaedal J., Jonsson P.V., Bjornsson S., Stefansson H., Sulem P., Gudbjartsson D., Maloney J. (2012). A mutation in APP protects against Alzheimer’s disease and age-related cognitive decline. Nature.

[173] Meng W.D., Sun S.J., Yang J., Chu R.X., Tu W., Liu Q. (2017). Elevated serum brain-derived neurotrophic factor (BDNF) but not BDNF gene Val66Met polymorphism is associated with autism spectrum disorders. Mol. Neurobiol..

[174] Bryn V., Halvorsen B., Ueland T., Isaksen J., Kolkova K., Ravn K., Skjeldal O.H. (2015). Brain derived neurotrophic factor (BDNF) and autism spectrum disorders (ASD) in childhood. Eur. J. Paediatr. Neurol..

[175] Li G.D., Bi R., Zhang D.F., Xu M., Luo R., Wang D., Fang Y., Li T., Zhang C., Yao Y.G. (2017). Female-specific effect of the BDNF gene on Alzheimer’s disease. Neurobiol. Aging.

[176] Amidfar M., de Oliveira J., Kucharska E., Budni J., Kim Y.K. (2020). The role of CREB and BDNF: Neurobiology and treatment of Alzheimer’s disease. Life Sci..

[177] Xie J., Huang L., Li X., Li H., Zhou Y., Zhu H., Pan T., Kendrick K.M., Xu W. (2017). Immunological cytokine profiling identifies TNF-α as a key molecule dysregulated in autistic children. Oncotarget.

[178] Zheng C., Zhou X.W., Wang J.Z. (2016). The dual roles of cytokines in Alzheimer’s disease: Update on interleukins, TNF-α, TGF-β and IFN-γ. Transl. Neurodegener..

[179] Calabrò M., Mandelli L., Crisafulli C., Porcelli S., Albani D., Politis A., Papadimitriou G.N., Di Nicola M., Janiri L., Colombo R. (2020). Psychiatric disorders and SLC6A4 gene variants: Possible effects on alcohol dependence and alzheimer’s disease. Mol. Biol. Rep..

[180] Ma D.Q., Rabionet R., Konidari I., Jaworski J., Cukier H.N., Wright H.H., Abramson R.K., Gilbert J.R., Cuccaro M.L., Pericak-Vance M.A. (2010). Association and gene–gene interaction of SLC6A4 and ITGB3 in autism. Am. J. Med Genet. Part B Neuropsychiatr. Genet..

[181] Courchesne E., Mouton P.R., Calhoun M.E., Semendeferi K., Ahrens-Barbeau C., Hallet M.J., Barnes C.C., Pierce K. (2011). Neuron number and size in prefrontal cortex of children with autism. JAMA.

[182] Courchesne E., Pierce K., Schumann C.M., Redcay E., Buckwalter J.A., Kennedy D.P., Morgan J. (2007). Mapping early brain development in autism. Neuron.

[183] Marchetto M.C., Belinson H., Tian Y., Freitas B.C., Fu C., Vadodaria K., Beltrao-Braga P.C., Trujillo C.A., Mendes A.P., Padmanabhan K. (2017). Altered proliferation and networks in neural cells derived from idiopathic autistic individuals. Mol. Psychiatry.

[184] Segawa M. (2005). Early motor disturbances in Rett syndrome and its pathophysiological importance. Brain Dev..

[185] Xu X., Miller E.C., Pozzo-Miller L. (2014). Dendritic spine dysgenesis in Rett syndrome. Front. Neuroanat..

[186] Rangasamy S., Olfers S., Gerald B., Hilbert A., Svejda S., Narayanan V. (2016). Reduced neuronal size and mTOR pathway activity in the Mecp2 A140V Rett syndrome mouse model. F1000Research.

[187] Winden K.D., Ebrahimi-Fakhari D., Sahin M. (2018). Abnormal mTOR Activation in Autism. Ann. Rev. Neurosci..

[188] Onore C., Yang H., Van de Water J., Ashwood P. (2017). Dynamic Akt/mTOR signaling in children with autism spectrum disorder. Front. Pediatrics.

[189] Busche M.A., Konnerth A. (2016). Impairments of neural circuit function in Alzheimer’s disease. Philos. Trans. R. Soc. B Biol. Sci..

[190] Dziewczapolski G., Glogowski C.M., Masliah E., Heinemann S.F. (2009). Deletion of the α7 nicotinic acetylcholine receptor gene improves cognitive deficits and synaptic pathology in a mouse model of Alzheimer’s disease. J. Neurosci..

[191] Gabriele S., Sacco R., Persico A.M. (2014). Blood serotonin levels in autism spectrum disorder: A systematic review and meta-analysis. Eur. Neuropsychopharmacol..

[192] Murphy D.L., Lesch K.P. (2008). Targeting the murine serotonin transporter: Insights into human neurobiology. Nat. Rev. Neurosci..

[193] Bonnin A., Goeden N., Chen K., Wilson M.L., King J., Shih J.C., Blakely R.D., Deneris E.S., Levitt P. (2011). A transient placental source of serotonin for the fetal forebrain. Nature.

[194] Lanari A., Amenta F., Silvestrelli G., Tomassoni D., Parnetti L. (2006). Neurotransmitter deficits in behavioural and psychological symptoms of Alzheimer’s disease. Mech. Ageing Dev..

[195] Previc F.H. (2006). The role of the extrapersonal brain systems in religious activity. Conscious. Cogn..

[196] Delaveau P., Salgado-Pineda P., Wicker B., Micallef-Roll J., Blinm O. (2005). Effect of levodopa on healthy volunteers’ facial emotion perception: An FMRI study. Clin. Neuropharmacol..

[197] Bachevalier J., Loveland K.A. (2006). The orbitofrontal-amygdala circuit and self-regulation of social–emotional behavior in autism. Neurosci. Biobehav. Rev..

[198] Pan X., Kaminga A.C., Wen S.W., Wu X., Acheampong K., Liu A. (2019). Dopamine and dopamine receptors in Alzheimer’s disease: A systematic review and network meta-analysis. Front. Aging Neurosci..

[199] Nam E., Derrick J.S., Lee S., Kang J., Han J., Lee S.J., Chung S.W., Lim M.H. (2018). Regulatory activities of dopamine and its derivatives toward metal-free and metal-induced amyloid-β aggregation, oxidative stress, and inflammation in Alzheimer’s disease. ACS Chem. Neurosci..

[200] Wang R., Reddy P.H. (2017). Role of glutamate and NMDA receptors in Alzheimer’s disease. J. Alzheimers Dis..

[201] Eltokhi A., Santuy A., Merchan-Perez A., Sprengel R. (2021). Glutamatergic dysfunction and synaptic ultrastructural alterations in schizophrenia and autism spectrum disorder: Evidence from human and rodent studies. Int. J. Mol. Sci..

[202] Srivastava A., Das B., Yao A.Y., Yan R. (2020). Metabotropic glutamate receptors in Alzheimer’s disease synaptic dysfunction: Therapeutic opportunities and hope for the future. J. Alzheimers Dis..

[203] Khlebodarova T.M., Kogai V.V., Trifonova E.A., Likhoshvai V.A. (2018). Dynamic landscape of the local translation at activated synapses. Mol. Psychiatry.

[204] Nordahl C.W., Scholz R., Yang X., Buonocore M.H., Simon T., Rogers S., Amaral D.G. (2012). Increased rate of amygdala growth in children aged 2 to 4 years with autism spectrum disorders: A longitudinal study. Arch. Gen. Psychiatry.

[205] Courchesne E. (2002). Abnormal early brain development in autism. Mol. Psychiatry.

[206] Alexiou A., Soursou G., Yarla N.S., Ashraf G.M. (2018). Proteins commonly linked to autism spectrum disorder and Alzheimer’s disease. Curr. Protein Pept. Sci..

[207] Mameza M.G., Dvoretskova E., Bamann M., Hönck H.H., Güler T., Boeckers T.M., Schoen M., Verpelli C., Sala C., Barsukov I. (2013). SHANK3 gene mutations associated with autism facilitate ligand binding to the Shank3 ankyrin repeat region. J. Biol. Chem..

[208] Bucher M., Niebling S., Han Y., Molodenskiy D., Kreienkamp H.J., Svergun D., Kim E., Kostyukova A.S., Kreutz M.R., Mikhaylova M. (2021). Autism associated SHANK3 missense point mutations impact conformational fluctuations and protein turnover at synapses. bioRxiv.

[209] Jaber V., Zhao Y., Lukiw W.J. (2017). Alterations in micro RNA-messenger RNA (miRNA-mRNA) coupled signaling networks in sporadic Alzheimer’s disease (AD) hippocampal CA1. J. Alzheimers Dis. Parkinsonism.

[210] Sokol D.K., Maloney B., Long J.M., Ray B., Lahiri D.K. (2011). Autism, Alzheimer disease, and fragile X: APP, FMRP, and mGluR5 are molecular links. Neurology.

[211] Steinberg J., Webberm C. (2013). The roles of FMRP-regulated genes in autism spectrum disorder: Single-and multiple-hit genetic etiologies. Am. J. Hum. Genet..

[212] Borreca A., Gironi K., Amadoro G., Ammassari-Teule M. (2016). Opposite dysregulation of fragile-X mental retardation protein and heteronuclear ribonucleoprotein C protein associates with enhanced APP translation in Alzheimer disease. Mol. Neurobiol..

[213] Barone R., Fichera M., De Grandi M., Battaglia M., Lo Faro V., Mattina T., Rizzo R. (2017). Familial 18q12. 2 deletion supports the role of RNA-binding protein CELF4 in autism spectrum disorders. Am. J. Med. Genet..

[214] McLane R.D., Schmitt L.M., Pedapati E.V., Shaffer R.C., Dominick K.C., Horn P.S., Gross C., Erickson C.A. (2019). Peripheral amyloid precursor protein derivative expression in fragile X syndrome. Fronti. Integr. Neurosci..

[215] Patel N., Hoang D., Miller N., Ansaloni S., Huang Q., Rogers J.T., Lee J.C., Saunders A.J. (2008). MicroRNAs can regulate human APP levels. Mol. Neurodegener..

[216] Chopra N., Wang R., Maloney B., Nho K., Beck J.S., Pourshafie N., Niculescu A., Saykin A.J., Rinaldi C., Counts S.E. (2020). MicroRNA-298 reduces levels of human amyloid-β precursor protein (APP), β-site APP-converting enzyme 1 (BACE1) and specific tau protein moieties. Mol. Psychiatry.

[217] Ruberti F., Barbato C., Cogoni C. (2010). Post-transcriptional regulation of amyloid precursor protein by microRNAs and RNA binding proteins. Commun. Integr. Biol..

[218] Pantopoulos K. (2004). Iron metabolism and the IRE/IRP regulatory system: An update. Ann. N. Y. Acad. Sci..

[219] Zhou Z.D., Tan E.K. (2017). Iron regulatory protein (IRP)-iron responsive element (IRE) signaling pathway in human neurodegenerative diseases. Mol. Neurodegener..

[220] Lee E.K., Kim H.H., Kuwano Y., Abdelmohsen K., Srikantan S., Subaran S.S. (2010). hnRNP C promotes APP translation by competing with FMRP for APP mRNA recruitment to P bodies. Nat. Struct. Mol. Biol..

[221] Hsu P.J., Shi H., Zhu A.C., Lu Z., Miller N., Edens B.M., Ma Y.C., He C. (2019). The RNA-binding protein FMRP facilitates the nuclear export of N6-methyladenosine–containing mRNAs. J. Biol. Chem..

[222] Schepens B., Tinton S.A., Bruynooghe Y., Parthoens E., Haegman M., Beyaert R. (2007). A role for hnRNP C1/C2 and Unr in internal initiation of translation during mitosis. EMBO J..

[223] Westmark C.J., Maloney B., Alisch R.S., Sokol D.K., Lahiri D.K. (2020). FMRP Regulates the Nuclear Export of Adam9 and Psen1 mRNAs: Secondary Analysis of an N 6-Methyladenosine Dataset. Sci. Rep..

[224] Ray B., Maloney B., Sambamurti K., Karnati H.K., Nelson P.T., Greig N.H. (2020). Rivastigmine modifies the α-secretase pathway and potentially early Alzheimer’s disease. Transl. Psychiatry.

[225] Rice H.C., De Malmazet D., Schreurs A., Frere S., Van Molle I., Volkov A.N., Creemers E., Vertkin I., Nys J., Ranaivoson F.M. (2019). Secreted amyloid-β precursor protein functions as a GABABR1a ligand to modulate synaptic transmission. Science.

[226] Tang B.L. (2019). Amyloid precursor protein (APP) and GABAergic neurotransmission. Cells.

[227] Ray B., Long J.M., Sokol D.K., Lahiri D.K. (2011). Increased secreted amyloid precursor protein-α (sAPPα) in severe autism: Proposal of a specific, anabolic pathway and putative biomarker. PLoS ONE.

[228] Zeng K., Kang J., Ouyang G., Li J., Han J., Wang Y., Sokhadze E.M., Casanova M.F., Li X. (2017). Disrupted brain network in children with autism spectrum disorder. Sci. Rep..

[229] Ray B., Sokol D.K., Maloney B., Lahiri D.K. (2016). Finding novel distinctions between the sAPPα-mediated anabolic biochemical pathways in Autism Spectrum Disorder and Fragile X Syndrome plasma and brain tissue. Sci Rep..

[230] Sokol D.K., Maloney B., Westmark C.J., Lahiri D.K. (2019). Novel contribution of secreted amyloid-β precursor protein to white matter brain enlargement in autism spectrum disorder. Front. Ppsychiatry.

